# Multitask connected U-Net: automatic lung cancer segmentation from CT images using PET knowledge guidance

**DOI:** 10.3389/frai.2024.1423535

**Published:** 2024-08-23

**Authors:** Lu Zhou, Chaoyong Wu, Yiheng Chen, Zhicheng Zhang

**Affiliations:** ^1^Traditional Chinese Medicine (Zhong Jing) School, Henan University of Chinese Medicine, Zhengzhou, Henan, China; ^2^Shenzhen Hospital, Beijing University of Chinese Medicine, Shenzhen, Guangdong, China; ^3^Shenzhen Institute of Advanced Technology, Chinese Academy of Sciences, Shenzhen, China

**Keywords:** lung cancer, CT image, PET/CT, medical image segmentation, deep learning

## Abstract

Lung cancer is a predominant cause of cancer-related mortality worldwide, necessitating precise tumor segmentation of medical images for accurate diagnosis and treatment. However, the intrinsic complexity and variability of tumor morphology pose substantial challenges to segmentation tasks. To address this issue, we propose a multitask connected U-Net model with a teacher-student framework to enhance the effectiveness of lung tumor segmentation. The proposed model and framework integrate PET knowledge into the segmentation process, leveraging complementary information from both CT and PET modalities to improve segmentation performance. Additionally, we implemented a tumor area detection method to enhance tumor segmentation performance. In extensive experiments on four datasets, the average Dice coefficient of 0.56, obtained using our model, surpassed those of existing methods such as Segformer (0.51), Transformer (0.50), and UctransNet (0.43). These findings validate the efficacy of the proposed method in lung tumor segmentation tasks.

## 1 Introduction

Despite significant advancements in diagnosing and treating lung cancer in recent decades, it remains the leading cause of cancer-related mortality globally, particularly among males (Sung et al., 2021). The introduction of low-dose computed tomography (CT) based lung cancer screening has notably reduced the mortality in clinical settings Leiter et al. (2023). This is mainly due to the advantages of CT's high spatial resolution and acceptable economic burden, making it widely used in the clinical setting. However, the limited contrast between malignant and non-malignant lesions in lung tissue, makes timely detection and accurate segmentation of cancer boundaries on CT images a challenge. This difficulty is further compounded by variations in tumor locations, intensities, shapes, and attachments to adjacent structures, necessitating accurate lung tumor segmentation (Mercieca et al., 2021). The use of positron emission tomography/computed tomography (PET/CT) imaging has become essential in the diagnosis and staging of cancer, as it provides both functional information from PET images and anatomical localization from CT images (Baek et al., 2019). PET/CT has become integral in tumor management, aiding in diagnosis, staging, and follow-up (Bianconi et al., 2020). In particular, PET/CT is crucial for early differentiation of benign and malignant tumors as well as disease severity assessment and progression. While CT images provide excellent spatial resolution, PET adds metabolic insights, allowing for superior tumor characterization. This further optimizes the screening and evaluation strategy for lung cancer; however, the high cost of PET and greater radioactive harm, limits its use compared to CT.

In the segmentation strategy for lung cancer, traditional manual contouring of boundaries on CT images is a time-consuming task prone to interobserver variability and misinterpretation. With the development and application of artificial intelligence (AI) technology, realizing the automatic segmentation of lung cancer based on CT images has become possible under the guidance of PET. Automatic tumor segmentation based on PET/CT images performs better than CT images alone (Ju et al., 2015). These automated segmentation methods largely focus on fusing the information extracted separately from the PET and CT modalities, under the assumption that each modality contains complementary information (Li et al., 2019; Bourigault et al., 2021; Cai et al., 2023). While acknowledging the advancements in PET/CT for lung cancer segmentation, addressing the associated drawbacks, such as the higher cost and increased radiation exposure to patients compared to CT alone has become crucial (Sheikhbahaei et al., 2017).

In this context, we explore the current status and challenges in lung cancer segmentation and highlight the evolving role of AI, particularly in the integration of PET and CT modalities for more accurate and comprehensive tumor segmentation. To mitigate the PET/CT concerns of higher cost and increased radiation exposure to patients compared with CT alone (Sheikhbahaei et al., 2017) and enhance the economic viability of lung cancer segmentation, we propose a novel deep learning-based connected U-Net model. This model aims to automatically fuse multimodal information by generating pseudo-PET images from CT images. The motivation behind this approach is to retain the benefits of PET-guided segmentation while minimizing the economic burden and radiation damage associated with PET examinations. Our contributions include:

1. **Multitask modeling framework:** A connected U-Net model and teacher-student framework are presented. In this framework, the model can learn two tasks: PET generation and tumor segmentation. This modeling approach allows for lung tumor segmentation guided by learned PET knowledge, eliminating the requirement for actual PET images. This simplifies the process of tumor segmentation, making it more practical and effective in the delineation of tumor boundary.
2. **Tumor area detection method:** To enhance the precision of tumor area delineation, we propose a tumor area detection method that enables more accurate segmentation by focusing on the area of the tumor.

This study mainly consists of six parts. **Part 1**, covers related work, presents a literature review of the field, and has been the inspiration for our research. **Part 2**, introduces the datasets and catalogs the public data sources utilized for training and validation. **Part 3** focuses on the methods, elucidating the architecture of multitask connected U-Net model and the semi-supervised learning based on our teacher-student framework and the proposed tumor area detection method. **Part 4**, provides the experimental settings and details of the technical environment and hyperparameters. **Part 5**, summarizes the results and presents a discussion on the model's performance across various metrics and datasets. **Part 6**, is the conclusion, providing a synthesis of the findings and suggesting future directions.

## 2 Related work

In recent years, significant advancements have been made in the field of medical image segmentation. These advancements have been primarily driven by the development of novel architectures and methodologies, which have significantly improved the performance of medical image segmentation, providing valuable tools for screening, clinical diagnosis, and treatment planning of lung cancer.

### 2.1 U-Net architecture

The U-Net architecture, presented by Ronneberger et al. (2015), has been a cornerstone in medical image segmentation. The architecture, consisting of a contracting path to capture context and a symmetric expanding path to enable precise localization, can be trained end-to-end on very few images and has demonstrated superior performance on several benchmarks. Several extensions and improvements to the U-Net architecture have been proposed. For instance, nnU-Net, a deep learning-based segmentation method, automatically configures itself for any new task, reducing the need for manual hyperparameter tuning (Isensee et al., 2021; Ferrante et al., 2022). Unet 3+ extends the U-Net architecture by incorporating full-scale skip connections, allowing better feature representation and more accurate segmentation results (Huang et al., 2020). UNeXt, a novel medical image segmentation network based on a convolutional multilayer perceptron (MLP), significantly reduces the number of parameters and decreases computational complexity compared to existing methods (Valanarasu and Patel, 2022). Zhang G. et al. (2022) proposed an improved 3D dense connected UNet (I-3D DenseUNet) for lung cancer segmentation from CT images. The nested dense skip connection adopted in the I-3D DenseUNet aims to contribute similar feature maps to the encoder and decoder sub-networks, encouraging feature propagation and reuse. U-Net's advantages include efficient biomedical image processing, an encoder-decoder structure for capturing context and details, skip connections for enhanced spatial coherence, strong performance with limited data, and adaptability to various medical imaging tasks, achieving state-of-the-art results.

### 2.2 Based on transformer architecture

The transformer architecture, originally proposed for natural language processing tasks, has also been widely adapted for medical image segmentation. UCTransNet presents a new segmentation framework, which uses a CTrans module to replace the original U-Net skip connection and conducts multiscale channel-wise fusion using the Transformer (Wang et al., 2022). TransUNet combines the strengths of the Transformer and U-Net, with the Transformer component encoding tokenized image patches from a convolutional neural network (CNN) feature map as the input sequence for extracting global contexts (Chen et al., 2021). SegFormer unifies the Transformer with the lightweight MLP decoder and includes a novel hierarchically structured Transformer encoder that outputs multiscale features (Xie et al., 2021). Recently, Tyagi et al. (2023) proposed an approach for lung cancer segmentation using an amalgamation of the vision transformer and CNN, demonstrating strong performance in lung cancer segmentation.

### 2.3 Cross-modality and teacher-student framework

Cross-modality learning, where information from one modality (e.g., PET) is used to enhance another modality (e.g., CT), has received increasing attention in recent years. The complementarity between PET and CT images allows the two modality images to be fused for automatic lung tumor segmentation. Zhang X. et al. (2022) proposed a network, based on two modality-specific encoders and two modality-specific decoders, that can fuse the complementary information and preserve modality-specific features of PET and CT images. In a similar vein, Bi et al. (2021) introduced a recurrent fusion network for multimodality PET/CT tumor segmentation, which iteratively fuses complementary image features from PET and CT images to refine segmentation results. These studies emphasize the simultaneous use of CT and PET medical images as input, to achieve information integration. However, there are some common concerns in PET scans, such as the high cost and usage of radioactive tracers among others, leading to a lack of PET data.

The teacher-student framework has been widely used in various fields and encompasses two design concepts, namely, knowledge distillation and semi-supervised learning. In the context of knowledge distillation, Hinton et al. (2015) demonstrated its effectiveness in compressing large models into smaller ones without significant loss of accuracy. For semi-supervised learning, Yu et al. (2019) presented a novel uncertainty-aware semi-supervised framework for left atrium segmentation from MR images. This framework consists of a student model and a teacher model, whereby the student model learns from the teacher model by minimizing segmentation and consistency losses with respect to the targets of the teacher model.

In this study, inspired by tumor segmentation using combined PET and CT images as well as the teacher-student framework in semi-supervised learning, we designed our teacher-student framework and proposed a connected U-Net model to integrate PET and CT knowledge. Utilizing the teacher-student framework, our model simultaneously learns the PET generation and tumor segmentation tasks, thereby leveraging the learned PET knowledge to generate pseudo-PET information, which is used in the segmentation process to eliminate the requirement for actual PET images and enhance segmentation performance.

## 3 Datasets

In this study, we used datasets from four public sources as shown in Table 1. These sources were: (1) NSCLC+Radiomics (NSCLC+Rad, updated 2021/06/01) (Bakr et al., 2018), (2) NSCLC-Radiomics (NSCLC-Rad, updated 2020/10/22), (3) NSCLC-Radiomics-Interobserver1 (NSCLC-Rad-Int, updated 2020/08/13), (4) Medical Segmentation Decathlon Task06 (MSD Task06) (Simpson et al., 2019; Antonelli et al., 2022). Among these four datasets, NSCLC + Rad has PET and CT images along with CT images with lung cancer segmentation labels; however, the segmentation labels do not correspond to PET images. The data of NSCLC-Rad comprise the largest number of CT images with lung cancer segmentation labels, but do not include PET images. The NSCLC-Rad-Int dataset also only includes CT images and segmentation labels, but each image contains multiple segmentation labels from different experts, whereby the regions that they collectively agree upon have been characterized as tumorous. The MSD Task06 is a well-known NSCLC segmentation competition dataset that includes CT images and tumor segmentation labels, specifically used to evaluate the performance of models in lung cancer segmentation. The production of these datasets involved the participation of domain experts and underwent rigorous proofreading, ensuring their high data quality.

**Table 1:** Description of datasets.

**Dataset**	**Total studies**	**Inclusion studies**	**Inclusion studies and used**
NSCLC+Rad	211	126	Training (100)/Validation (13)/Test (13)
NSCLC-Rad	422	421	External Test (421)
MSD Task06	127	63	External Test (63)
NSCLC-Rad-Int	22	22	External Test (22)

In this study, only the NSCLC+Rad dataset was used for model training. We chose studies with CT images and corresponding PET images (Data A) or CT images with segmentation mask labels (Data B). After cleaning the data, 126 studies out of the original 211 in the NSCLC+Rad dataset were retained. These studies were divided as follows: 80% (100 studies) for training the model, 10% (13 studies) for validation, and the remaining 10% (13 studies) for testing the model. For external validation, we used three additional datasets with lung cancer mask labels, namely, NSCLC-Rad, NSCLC-Rad-Int, and MSD Task06, totaling 506 studies. To standardize the CT images across all datasets, we resized them to 512 × 512 with a pixel spacing of 1 mm using trilinear interpolation. Each image was further cropped to a size of 288 × 288 pixels to facilitate training on our devices.

## 4 Methods

### 4.1 Connected U-Net architecture

**Figure 1:**
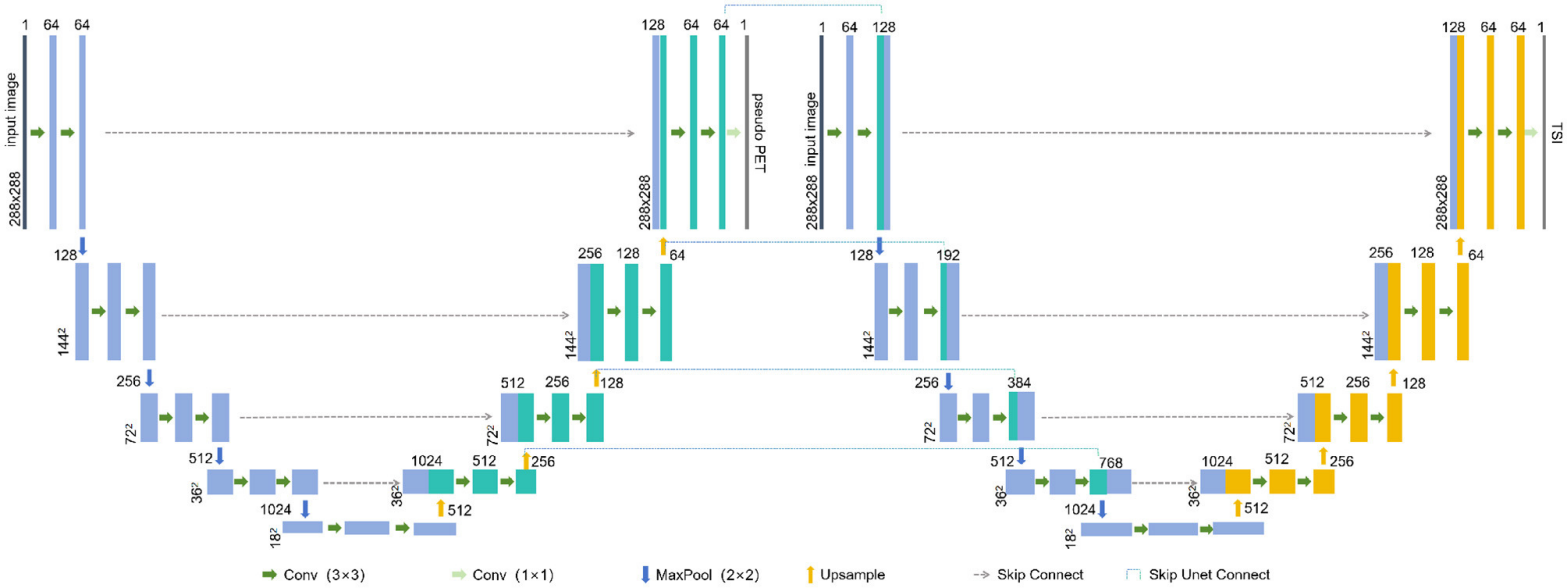
The connected U-Net architecture. TSI, tumor segmentation images.

**Figure 2:**
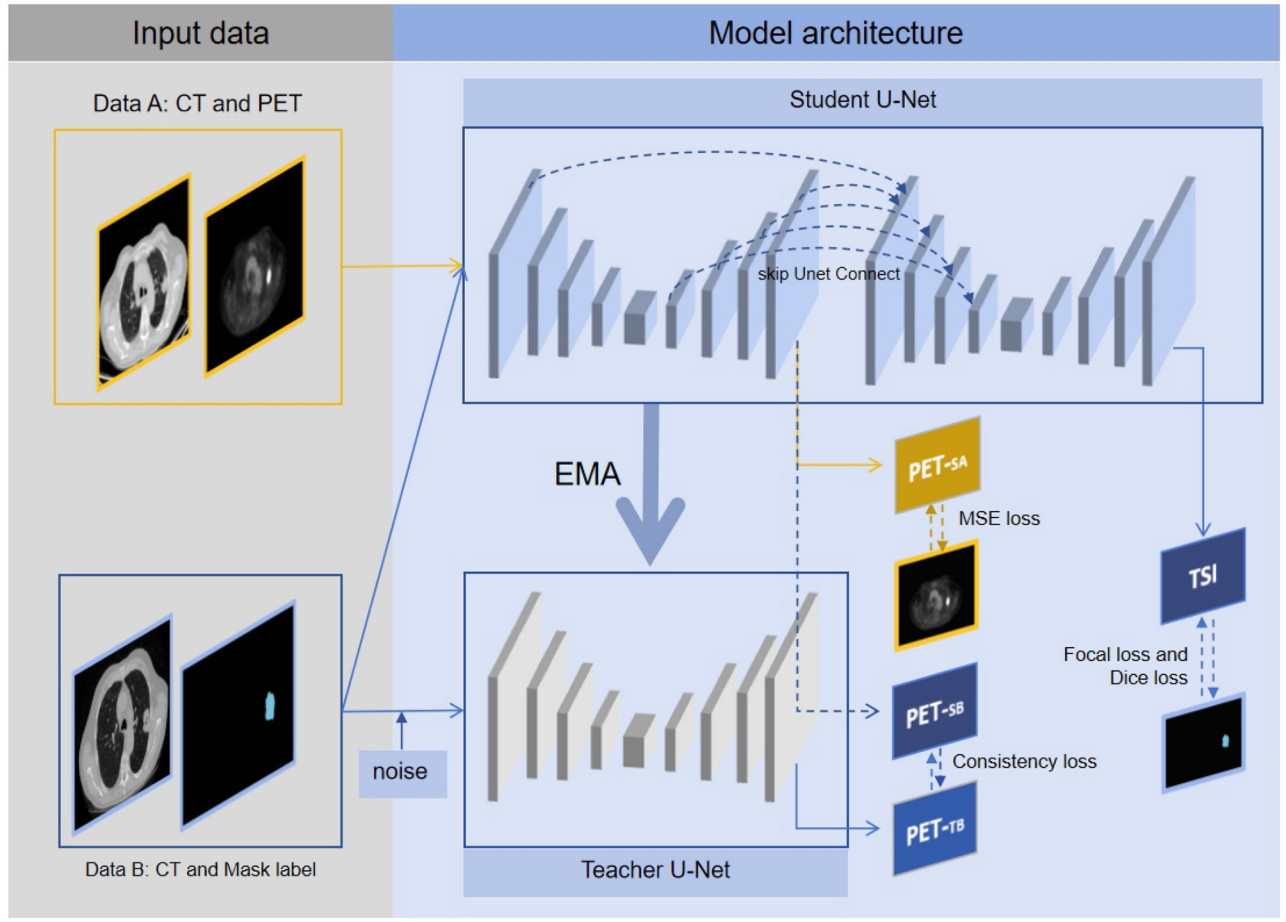
The multitask connected U-Net model with teacher-student framework. Data A are solely utilized for model construction, with the first U-Net in student U-Net generating pseudo PET_*SA*_, optimizing parameters through MSE loss, and updating parameters of teacher U-Net via EMA. Data B are fed into both student U-Net and teacher U-Net, with the first U-Net of the student U-Net generating pseudo PET_*SB*_ and the pseudo PET_*TB*_ produced by the teacher U-Net. Based on pseudo PET_*SB*_ and PET_*TB*_, consistency loss is obtained. Additionally, TSI is generated by the second U-Net of the student U-Net, with parameters optimized through focal, dice, and consistency losses. Through the above framework, the proposed segmentation model can simultaneously learn tumor segmentation and corresponding pseudo-PET generation, thereby integrating PET knowledge into the segmentation model.

The connected U-Net architecture is illustrated in Figure 1. In this dual U-Net model, the first U-Net generates pseudo-PET images, whereas the second U-Net produces tumor segmentation images (TSI). For the segmentation process, the features that are used to generate pseudo-PET images are obtained by upsampling the first U-Net and connected with the features obtained by downsampling the second U-Net, thereby incorporating the PET information into the segmentation process. As illustrated in Figure 2, we employ the teacher-student framework, which enables the proposed model to simultaneously learn the PET generation and tumor segmentation tasks, whereby the model can use the learned PET knowledge to generate pseudo-PET information, using it in the segmentation process, as shown in Figure 1.

In the teacher-student framework, Data A's CT images were fed into the student U-Net, resulting in the production of pseudo-PET_*SA*_ images. The quality of the pseudo-PET effects was assessed using mean-squared-error (MSE) loss. Concurrently, Data B's CT images were input into the student U-Net. This process yields pseudo-PET_*SB*_ from the first U-Net, whereas the second U-Net outputs a TSI. Additionally, the teacher U-Net processes the randomly rotated the Data B's CT images to generate pseudo-PET_*TB*_. The similarity between pseudo-PET_*SB*_ and pseudo-PET_*TB*_ was measured using consistency loss to evaluate the consistency of the model output. The tumor segmentation model performance was evaluated through focal and dice losses, to assess tumor identification and segmentation efficiency.

**First U-Net:** Each U-Net consisted of an input layer, an architecture of four downsampling (encoder) layers and four upsampling (decoder) layers. Each encoder architecture consisted of two 3 × 3 convolutions (each followed by a leaky rectified linear unit *LeakyReLU* and instance normalization operation) with padding set to 1. We define this convolution-based operation as *Conv*. After the convolutions, a 2 × 2 max pooling operation with a stride of 1 was employed for each downsampling step, which we define as *maxPool*. The decoder architecture consisted of a convolution operation and an upsampling operation followed by a 2 × 2 convolution, which we define as *UpSampling*. For the output of the first U-Net, a 1 × 1 convolution was used to map each feature vector to the pixel value of the PET, which we define as *outConv*. The down-sampling and up-sampling processes of the first U-Net can be represented as follows:

The input layer:


(1) $$\begin{array}{l} {\hat{x}}_{1}=Conv\left(LeakyReLU\left(x_{1}\right)\right) \end{array}$$

(2) $$\begin{array}{l} q_{1}=Conv\left(LeakyReLU\left({\hat{x}}_{1}\right)\right) \end{array}$$

Each down-sampling (encoder) layer:


(3) $$\begin{array}{l} x_{i}=maxPool\left(q_{i}\right) \end{array}$$

(4) $$\begin{array}{l} {\hat{x}}_{i}=Conv\left(LeakyReLU\left(x_{i}\right)\right) \end{array}$$

(5) $$\begin{array}{l} q_{i+1}=Conv\left(LeakyReLU\left({\hat{x}}_{i}\right)\right) \end{array}$$

Each up-sampling (decoder) layer:


(6) $$\begin{array}{l} u_{j}=skipConnect\left(q_{i},e_{j}\right) \end{array}$$

(7) $$\begin{array}{l} {û}_{j}=UpSampling\left(u_{j}\right) \end{array}$$

(8) $$\begin{array}{l} {ê}_{j}=Conv\left(LeakyReLU\left({û}_{j}\right)\right) \end{array}$$

(9) $$\begin{array}{l} e_{j+1}=Conv\left(LeakyReLU\left({ê}_{j}\right)\right) \end{array}$$

The output layer:


(10) $$\begin{array}{l} {ê}_{last}=Conv\left(LeakyReLU\left(e_{last}\right)\right) \end{array}$$

(11) $$\begin{array}{l} y_{pet}=OutputConv\left(Conv\left(LeakyReLU\left({ê}_{last}\right)\right)\right) \end{array}$$

In the Equations 1–11, *x*_*i*_ is the input of the *i*-th downsampling process. *x*_1_ is the CT image, and *u*_*j*_ is the input of the *j*-th upsampling process. The function *skipConnect* represents the concatenation of the corresponding feature map from upsampling to that from downsampling in the same U-Net.

**Second U-Net:** The second U-Net introduces a skip connection *skipUnetConnect* from the first U-Net's upsampling to its downsampling. This connection is defined as:


(12) $$\begin{array}{l} x_{i}=skipUnetConnect\left(e_{j},q_{i}\right) \end{array}$$

In the Equation 12, *skipUnetConnect* represents the concatenation of the corresponding feature map from the upsampling of the first U-Net to the downsampling of the second U-Net. *q*_*i*_ is the *i*-th downsampling feature of the second U-Net. *e*_*j*_ is the *j*-th upsampling feature of the first U-Net.

### 4.2 Semi-supervised multitask learning with teacher-student framework

PET images are more difficult to obtain than CT images. In our collected datasets, there were CT scans with segmentation labels but no PET images, and there were CT scans with PET images. To address this, we constructed a teacher-student framework to learn tumor segmentation and simultaneously learn pseudo-PET generation by semi-supervised learning, as illustrated in Figure 2. We denote Data A as *D*_*l*_ and Data B as *D*_*u*_. The training loss *L* is defined as follows:


(13) $$\begin{array}{r} L=w\frac{1}{\left|D_{l}\right|}\underset{x\in D_{l}}{\sum }C\left(f_{\theta }\left(x\right),f_{\theta^{\prime }}\left(x\right)\right)+\frac{1}{\left|D_{u}\right|}\underset{x,y_{pet}\in D_{u}}{\sum }H\left(y_{pet},f_{\theta }\left(x\right)\right)\\ +\frac{1}{\left|D_{l}\right|}\underset{x,y_{seg}\in D_{l}}{\sum }F\left(y_{seg},f_{\theta }\left(x\right)\right) \end{array}$$

In the Equation 13, *f*_θ_ represents the connected U-Net model, and the θ represent the model weights. $C\left(f_{\theta }\left(x\right);f_{\theta^{\prime }}\left(x\right)\right)$ denotes the consistency loss, with *w* as the loss coefficient. *H*(*y*_*pet*_, *f*_θ_(*x*)) represents the consistency loss of the actual PET and pseudo PET, with the consistency loss computed by MSE. *F*(*y*_*seg*_; *f*_θ_(*x*)) denotes the sum of the focal and dice losses of the segmentation mask label and TSI; $f_{\theta^{\prime }}$ represents the teacher U-Net. After training for *t* steps, θ′ is updated by exponential moving average (EMA):


(14) $$\begin{array}{l} \theta_{t}^{\prime }=\alpha \theta {\left(t-1\right)}^{\prime }+\left(1-\alpha \right)\theta_{t} \end{array}$$

In the Equations 13, 14, both *w* and α are dynamically adjusted according to:


(15) $$\begin{array}{l} \alpha =min\left(1-\frac{1}{step+1},0.99\right) \end{array}$$

The *w* is specified in Laine and Aila (2017) and expressed as:


(16) $$\begin{array}{l} w=0.1\times \exp\left(-5\times {\left(1-T\right)}^{2}\right) \end{array}$$

In the Equations 15, 16, T = 1- *step*/80 (Laine and Aila, 2017), where *step* refers to the number of training steps.

### 4.3 Tumor area detection preprocessing

**Figure 3:**
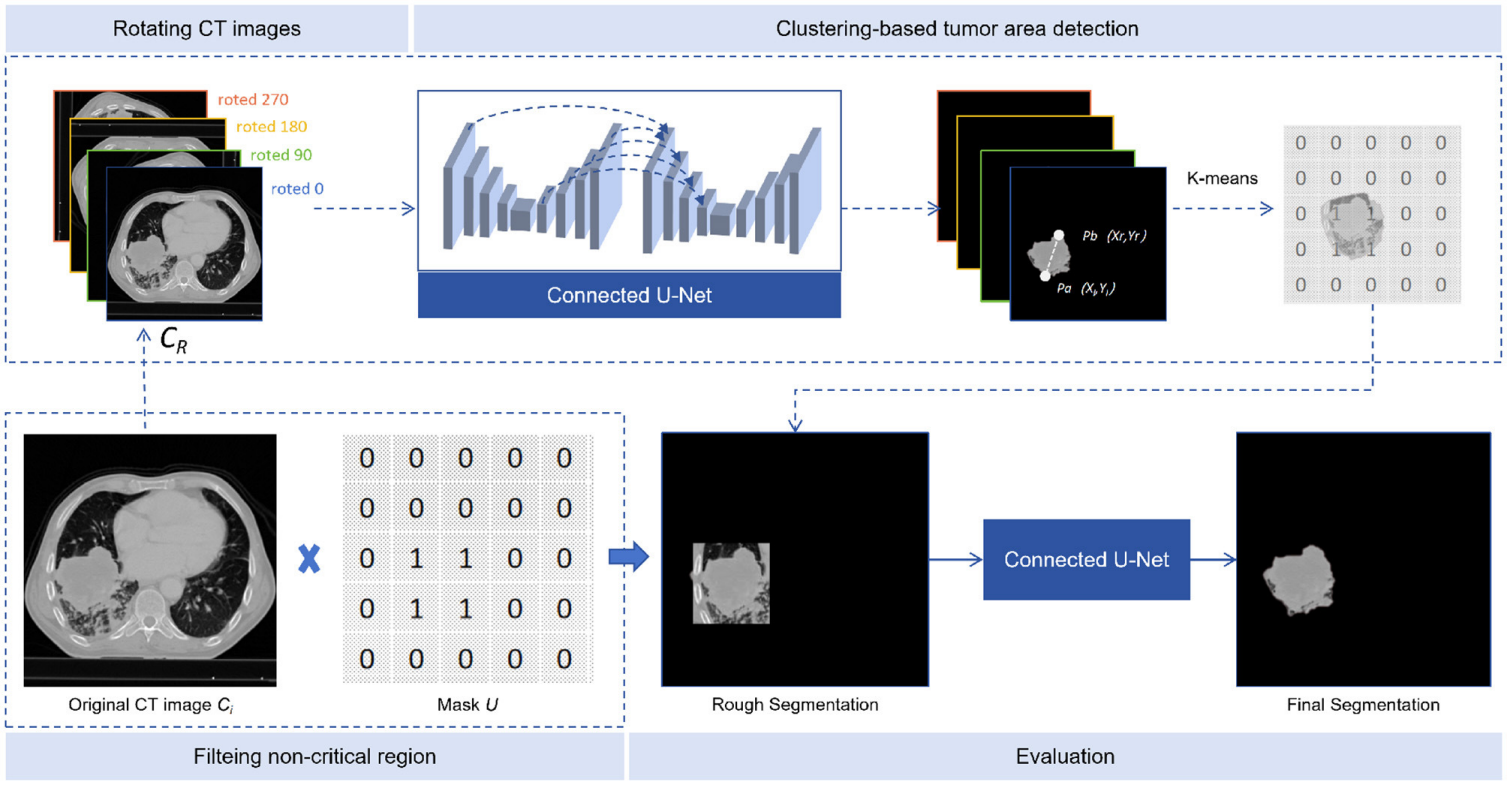
Tumor area detection. The tumor area detector consists of three steps: rotated CT slices, clustering-based tumor area detection, and filtering of non-critical regions.

Tumor area detection aims to filter out areas without tumors from CT images, enabling our model to concentrate on the segmentation of tumor areas. As shown in Figure 3, the process consists of three stages: rotating CT slices, clustering-based tumor area detection, and filtering non-critical regions.

#### 4.3.1 CT slices rotation

Defining *C*_*T*_ as the sequence of CT slices, where *C*_*i*_ is the *i*-th slice, each slice was rotated by α degrees (90, 180, and 270) to obtain three additional rotated slices. These were then inserted after the original slice, to form a new sequence *C*_*R*_, as shown in Equation 17:


(17) $$\begin{array}{l} C_{R}=\left(C_{0},C_{0}^{90},C_{0}^{180},C_{0}^{270},C_{1},C_{1}^{90},C_{1}^{180},C_{1}^{270},\ldots ,C_{i}^{\alpha }\right) \end{array}$$

#### 4.3.2 Clustering-based tumor area detection

The input of our model was *C*_*R*_ and the output TSI. In the multiple continuous TSI depicting the tumor boundary, the middle slice was selected as *S*_*N*_ and the 10 TSIs before and after it, together with the middle one, add up to 21 TSIs. On each selected TSI, points *p*_*i*_ and *p*_*j*_ can be represented as *p*_*i*_(*x*_*i*_, *y*_*i*_) and *p*_*j*_(*x*_*j*_, *y*_*j*_). The Euclidean distance between pixels is calculated as Equation 18:


(18) $$\begin{array}{l} d\left(p_{i},p_{j}\right)=\sqrt{{\left(x_{i}-x_{j}\right)}^{2}+{\left(y_{i}-y_{j}\right)}^{2}} \end{array}$$

We then find the two farthest points on each TSI: *P*_*a*_(*x*_*l*_, *y*_*l*_) and *P*_*b*_(*x*_*r*_, *y*_*r*_), and form a quadruple, as shown in Equation 19:


(19) $$\begin{array}{l} quadruple=\left(x_{l},y_{l},x_{r},y_{r}\right) \end{array}$$

All quadruples from TSIs are input into the K-means algorithm to obtain two cluster centers: *K*_1_ and *K*_2_.

#### 4.3.3 Non-critical area filtering

To compare the number of quadruples in *K*_1_ and *K*_2_, we selected the group with more quadruples, calculating the mean values of each column in the quadruplets to obtain two points *P*_0_ and $P_{0}^{\prime }$, thereby extending λ pixels (λ was set to 50 in our study) from the points obtained to create a rectangular region. A mask *U* was created, with the region set to 1 and the rest to 0. The critical region of each CT slice was found by applying the mask *U* to *C*_*i*_, and the masked image was re-predicted using the connected U-Net model, as shown in Equation 20:


(20) $$\begin{array}{l} y_{seg}=f_{\theta }\left(C_{i}\times U\right) \end{array}$$

## 5 Experimental settings

The connected U-Net model was constructed using Pytorch 1.13 and trained on an NVIDIA GeForce RTX 3080 Ti GPU 12 GB, Intel(R) Core(TM) i7-12700KF, RAM 16 GB. Throughout training, data augmentation techniques such as random rotation, random flipping, and random cropping were applied. The main hyperparameters were batch size, learning rate, and optimizer. Considering our hardware capability, the hyperparameter of batch size was set to 8. The learning rate of the hyperparameter was set to 1e-4, utilizing the Adam optimizer and employing a cosine learning rate control scheme (the change in learning rate for each epoch was from 1e-4 to 1e-5), to avoid the problem of falling into saddle points through the periodic change in the learning rate. The model that yielded the best evaluation results was selected for testing.

The model was evaluated using the dice similarity coefficient (DSC), intersection over union (IOU), and Hausdorff 95% (HD 95%) as test metrics. These metrics were calculated using the Medpy package. Additionally, we analyzed the number of parameters and the speed of our model using the thop package, comparing these values with those of other models such as Unet3+ (Huang et al., 2020), UNeXt (Valanarasu and Patel, 2022), UctransNet (Wang et al., 2022), TransUnet (Chen et al., 2021), and SegFormer (Xie et al., 2021).

## 6 Results and discussion

The integration of PET and CT modalities has revolutionized lung cancer segmentation, by providing more anatomical information for superior tumor location. Some studies emphasize the significance of leveraging PET and CT images as inputs for tumor segmentation, highlighting that integration of multimodal imaging can enhance performance outcomes (Alshmrani et al., 2023; Marinov et al., 2023; Zhou et al., 2023). However, drawbacks such as higher costs and increased radiation exposure compared to CT alone preclude the need for innovative solutions (Sheikhbahaei et al., 2017; Edelman Saul et al., 2020). In this context, our proposed connected U-Net model, combined with teacher-student semi-supervised multitask framework, emerges as a promising method, aiming to fuse pseudo-PET features for segmentation processing without the need for actual PET images.

### 6.1 Examples of model performance on different datasets

**Figure 4:**
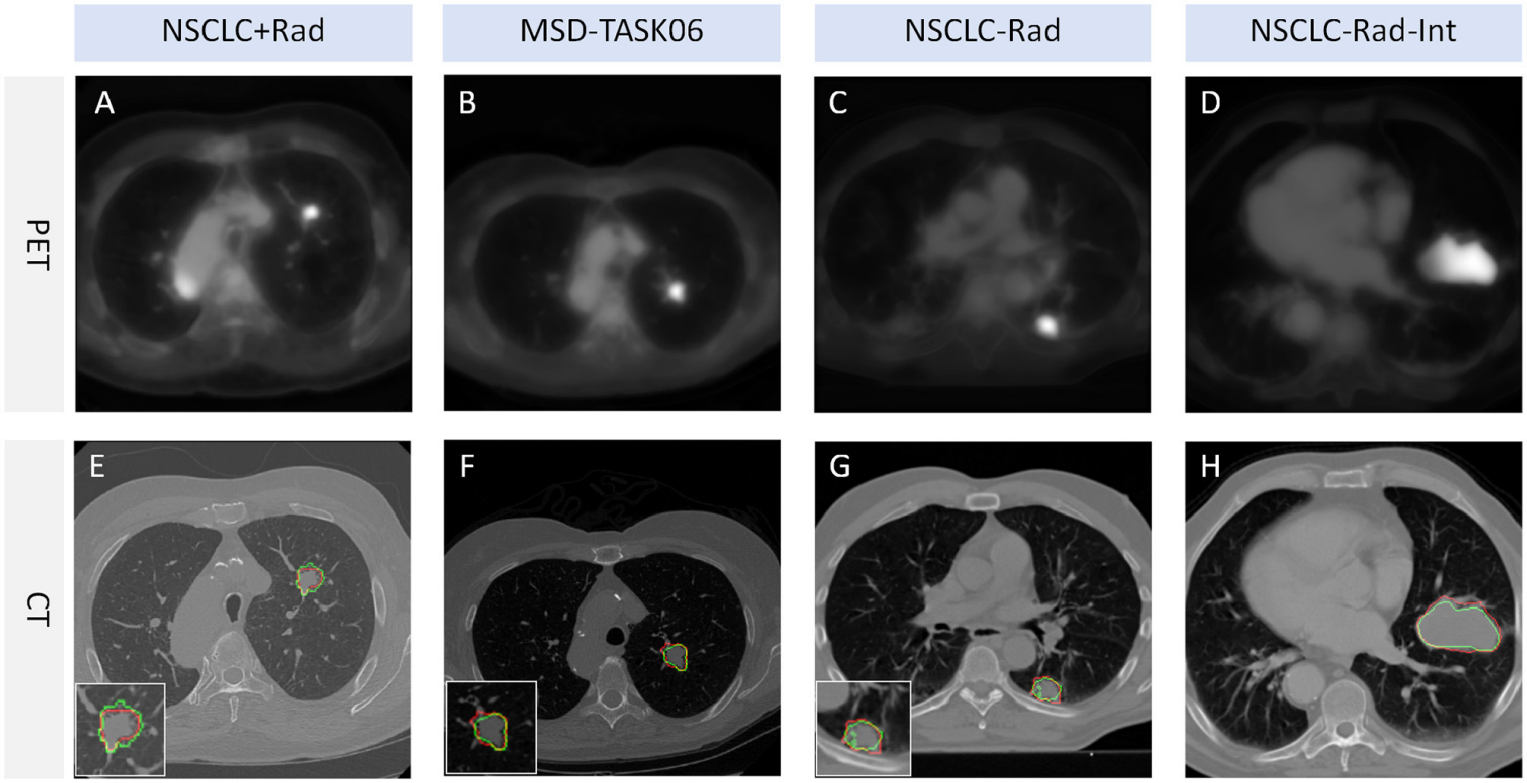
Tumor segmentation visualization. The red line represents the segmentation result of TSI, whereas the green line represents the mask label. TSI refers to the tumor segmentation image. **(A, E)** represent a lung cancer PET image and its corresponding CT image segmentation example from the NSCLC+Rad dataset, respectively. **(B, F)** represent a lung cancer PET image and its corresponding CT image segmentation example from the MSD-TASK06 dataset, respectively. **(C, G)** represent a lung cancer PET image and its corresponding CT image segmentation example from the NSCLC-Rad dataset, respectively. **(D, H)** represent a lung cancer PET image and its corresponding CT image segmentation example from the NSCLC-Rad-Int dataset, respectively.

Our model underwent testing on diverse datasets, demonstrating effective generalization capabilities. Guided by pseudo-PET and tumor area detection, the model excels in accurately pinpointing tumor growth areas. The model delivers impressive performance across various datasets, as illustrated in Figure 4. This result shows that the proposed model and framework do not mandate a correspondence between PET images and segmentation labels during training. Furthermore, the prediction stage also does not require PET images, suggesting promising avenues for practical applications and flexibility in real-world scenarios.

### 6.2 DSC of different models on different datasets

This study includes a series of experiments conducted on three public datasets, demonstrating the effectiveness of the proposed methods. Our model stands out among the evaluated models, showcasing remarkable adaptability across datasets. Notably, on the challenging MSD Task06 dataset, our model achieved a high DSC score of 0.66, surpassing competitor models, as detailed in Table 2. On the NSCLC+Rad dataset, our model achieved a DSC of 0.64, highlighting its effectiveness in the context of lung cancer segmentation. Moreover, on the NSCLC+Rad and NSCLC-Rad-Int datasets, our model achieved DSC of 0.64 and 0.57, respectively, highlighting its effectiveness in the context of lung cancer segmentation. While Segformer excels on the NSCLC+Rad dataset, our model matches and even surpasses its performance on the MSD Task06 dataset. These results indicate the proposed model's ability to adapt to diverse datasets, positioning it as a promising solution for a wide array of medical image segmentation tasks. Overall, our model not only competes effectively with existing models but also showcases reliability across varied medical imaging scenarios. The efficiency of the proposed model is evident in its moderate parameter count (36.605M) and FLOPs (50.716G), striking a balance between model complexity and segmentation accuracy. This underscores the practical applicability of our model in real-world medical image segmentation applications, offering a compelling blend of high performance and computational efficiency.

**Table 2:** DSC for different models on different datasets (mean ± SD).

**Model**	**Param**	**Flops**	**NSCLC + Rad**	**NSCLC-Rad-Int**	**MSD Task06**
Unet3+; Huang et al. (2020)	26.974M	199.856G	0.38 ± 0.31	0.29 ± 0.23	0.32 ± 0.30
UNeXt; Valanarasu and Patel (2022)	1.472M	0.554G	0.44 ± 0.30	0.25 ± 0.24	0.30 ± 0.30
UctransNet; Wang et al. (2022)	66.241M	54.386G	0.63 ± 0.29	0.32 ± 0.32	0.57 ± 0.32
TransUnet; Chen et al. (2021)	93.232M	40.812G	0.59 ± 0.30	0.43 ± 0.33	0.60 ± 0.31
Segformer; Xie et al. (2021)	84.595M	31.563G	**0.64** **±** **0.29**	0.46 ± 0.32	0.64 ± 0.29
Ours	36.605M	50.716G	**0.64** **±** **0.27**	**0.57** **±** **0.29**	**0.66** **±** **0.27**

DSC, Dice Similarity Coefficient. The bolded values represent the optimal evaluation metrics for different modeling strategies within the same dataset.

### 6.3 IOU and HD 95% of different models on different datasets

The proposed model stands out with outstanding segmentation performance across different datasets, particularly on the MSD Task06 dataset, as shown in Table 3. It achieves the highest IOU score of 0.55, showcasing superior accuracy in delineating segmented structures compared to other models. This result highlights the effectiveness of our model in capturing the intricate details of medical images. Furthermore, in terms of 95% HD, our model also excels with a notable score of 13.05, demonstrating its ability to precisely capture the boundaries of segmented regions. The performance of the proposed model surpasses or closely rivals other state-of-the-art models such as Segformer.

**Table 3:** IOU and HD95% of different models on different datasets.

**Dataset**	**Model**	**IOU(mean ± SD)**	**HD95%(mean ± SD)**
NSCLC+Rad	Unet3+	0.29 ± 0.26	116.21 ± 67.90
	UNeXt	0.33 ± 0.26	112.36 ± 74.89
	UctransNet	0.52 ± 0.27	57.60 ± 74.29
	TransUnet	0.48 ± 0.27	50.31 ± 59.95
	Segformer	**0.53** **±** **0.27**	36.14 ± 54.57
	Ours	0.52 ± 0.25	**18.23** **±** **32.25**
NSCLC-Rad-Int	Unet3+	0.19 ± 0.17	116.62 ± 49.44
	UNeXt	0.16 ± 0.18	112.29 ± 54.79
	UctransNet	0.24 ± 0.26	63.34 ± 54.06
	TransUnet	0.33 ± 0.28	63.79 ± 60.28
	Segformer	0.36 ± 0.28	37.94 ± 46.23
	Ours	**0.45** **±** **0.26**	**21.46** **±** **27.81**
MSD Task06	Unet3+	0.23 ± 0.25	133.64 ± 68.43
	UNeXt	0.22 ± 0.24	133.59 ± 73.64
	UctransNet	0.46 ± 0.29	38.83 ± 59.06
	TransUnet	0.50 ± 0.28	41.74 ± 58.51
	Segformer	0.53 ± 0.27	23.88 ± 45.08
	Ours	**0.55** **±** **0.26**	**13.05** **±** **23.27**

IOU, intersection over union; HD, Hausdorff distance. The bolded values represent the optimal evaluation metrics for different modeling strategies within the same dataset.

### 6.4 External validation on the challenge dataset

The NSCLC-Rad dataset has a larger sample size. On this dataset, all the models we used performed poorly. The performance of the model depends on various factors such as the quality of the images as well as the size and shape of the tumors (Tyagi et al., 2023). The poor performance is likely the result of distributional differences between this dataset and the training data. Nevertheless, our segmentation model, based on connected U-Net and tumor area detection, exhibits notable performance compared to other models, as shown in Table 4.

**Table 4:** Performance of different models on NSCLC-Rad.

**Model**	**Dice (mean ± SD)**	**IOU (mean ± SD)**	**HD95% (mean ± SD)**
Unet3+	0.23 ± 0.26	0.16 ± 0.20	105.02 ± 57.40
UNeXt	0.21 ± 0.27	0.15 ± 0.21	82.30 ± 61.12
UctransNet	0.23 ± 0.29	0.17 ± 0.23	75.52 ± 58.58
TransUnet	**0.38** **±** **0.33**	**0.29** **±** **0.27**	59.17 ± 55.07
Segformer	0.31 ± 0.32	0.23 ± 0.26	51.62 ± 49.76
Ours	**0.38** **±** **0.32**	**0.29** **±** **0.27**	**42.26** **±** **41.20**

DSC, Dice similarity coefficient; IOU, intersection over union; HD, Hausdorff distance. The bolded values represent the optimal evaluation metrics for different modeling strategies within the same dataset.

### 6.5 Subgroup analysis by slice and distance

Identifying small lesions in their early stages remains a challenging task. The size of the tumor significantly affects the performance of segmentation models. Coronal CT images allow the measurement of the long and short diameters of tumors and evaluation of their area. Additionally, continuous CT scans enable the calculation of tumor height in the sagittal plane based on layer thickness and the number of scanned layers, which can estimate tumor volume.

**Figure 5:**
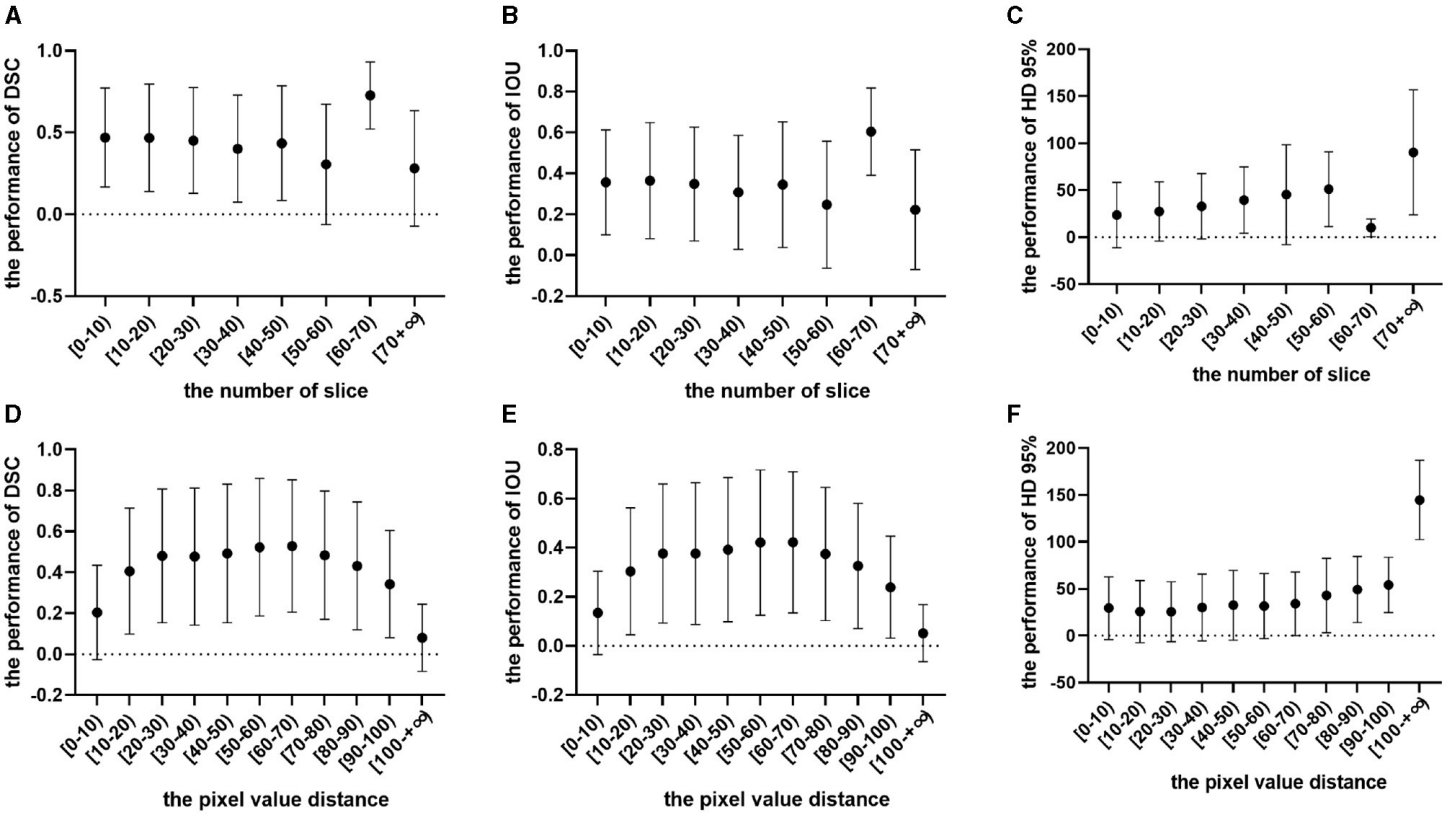
Effects of slice number and distance. **(A)** Represents the Dice similarity coefficient (DSC) performance within a specific subgroup of a slice. **(B)** Denotes the intersection over union (IOU) performance within the same subgroup of a slice. **(C)** Indicates the Hausdorff distance (HD) at 95% performance within the subgroup of a slice. **(D)** Signifies the DSC performance within a specific subgroup of a distance. **(E)** Represents the IOU performance within the same subgroup of a distance. **(F)** Denotes the HD at 95% performance within the subgroup of a distance.

In our investigation, subgroup analyses were conducted using slice thickness and distance to explore the impact of tumor size on model segmentation across various datasets, as shown in Figure 5. Unexpectedly, the slice number was found to have no significant impact on tumor segmentation. In further analysis, the intermediate CT section yielded the best segmentation results when dealing with multiple slices containing tumor CT. Notably, patient chest CT scans from different sources may exhibit varying slice thicknesses, leading to potential errors in estimating tumor height based on slice number.

Furthermore, the long diameter of the tumor can be represented by the pixel value distance. As the tumor pixel value distance increases, the model' s segmentation performance improves significantly. However, beyond a certain threshold, further expansion of the tumor pixel value may lead to a decline in segmentation effectiveness. This phenomenon could be attributed to limited modeling data and a lack of training samples for giant tumors.

### 6.6 Ablation experiment

The teacher-student framework allows our proposed model to concurrently learn PET generation and tumor segmentation tasks. Through this approach, our proposed model can use the learned PET knowledge to generate pseudo-PET information, subsequently integrating the information into segmentation process. To verify the effectiveness of integrating pseudo-PET information, we tested it on four datasets through ablation experiments, as outlined in Table 5. The results showed that our model, when integrating pseudo-PET images, achieved better Dice values on the NSCLC+Rad dataset (0.64 vs 0.59) than the model based solely on CT images. In addition, the average of the HD 95% results on the four datasets (37.65 vs 43.55) also indicates that the model based on guidance from learned PET knowledge exhibits better performance in the processing of edge details for tumor boundaries.

**Table 5:** Performance of multitask connected U-Net based on different components.

**Dataset**	**Model**	**Dice (mean ± SD)**	**IOU (mean ± SD)**	**HD95% (mean ± SD)**
NSCLC+Rad	Only based on CT images	0.59 ± 0.29	0.48 ± 0.27	44.79 ± 57.82
	PET guide	**0.64** **±** **0.29**	**0.53** **±** **0.26**	28.57 ± 49.11
	PET guide and area detection	**0.64** **±** **0.27**	0.52 ± 0.25	**18.23** **±** **32.25**
NSCLC-Rad	Only based on CT images	0.35 ± 0.33	0.27 ± 0.28	54.74 ± 54.15
	PET guide	0.24 ± 0.28	0.17 ± 0.22	62.21 ± 57.18
	PET guide and area detection	**0.38** **±** **0.32**	**0.29** **±** **0.27**	**42.26** **±** **41.20**
NSCLC-Rad-Int	Only based on CT images	0.51 ± 0.33	0.40 ± 0.29	44.40 ± 52.61
	PET guide	0.51 ± 0.32	0.40 ± 0.28	32.99 ± 47.15
	PET guide and area detection	**0.57** **±** **0.29**	**0.45** **±** **0.27**	**21.46** **±** **27.81**
MSD Task06	Only based on CT images	0.65 ± 0.30	0.54 ± 0.28	30.28 ± 53.86
	PET guide	0.63 ± 0.30	0.52 ± 0.28	26.83 ± 49.49
	PET guide and area detection	**0.66** **±** **0.27**	**0.55** **±** **0.26**	**13.05** **±** **23.27**
Average	Only based on CT images	0.53 ± 0.31	0.42 ± 0.28	43.55 ± 54.61
	PET guide	0.51 ± 0.30	0.41 ± 0.26	37.65 ± 50.73
	PET guide and area detection	**0.56** **±** **0.29**	**0.45** **±** **0.26**	**23.75** **±** **31.13**

DSC, Dice similarity coefficient; IOU, intersection over union; HD, Hausdorff distance. The bolded values represent the optimal evaluation metrics for modeling models based on different.

Tumor area detection aims to filter out areas without tumors from CT images, enabling the established model to further focus on segmenting only the areas where tumors are present. The results showed that this approach further enhanced the model's performance on the NSCLC-Rad and NSCLC-Rad-Int datasets, reaffirming the generalizability and effectiveness of the proposed enhancements. The MSD Task06 dataset similarly reflects consistent improvement, culminating in the best overall performance when our model utilizes learned PET knowledge and applies tumor area detection. These shared trends underscore the efficacy of the proposed methods across various datasets, reinforcing their potential for enhancing medical image segmentation tasks.

## 7 Limitation

Although, our model aimed to alleviate the economic burden and radiation risks associated with PET examinations, extensive research is required to evaluate the long-term cost-effectiveness and safety implications of this approach. Continued investigations will be crucial in assessing the viability and sustainability of implementing our proposed methodology. Moreover, the tumor area detection in this study is constrained to single tumors, warranting validation for its effectiveness in localizing multiple tumors. This aspect requires further verification to ensure the model's applicability to scenarios involving multiple tumor instances.

## 8 Conclusion

In this study, we proposed a multitask connected U-Net model and a teacher-student framework. The framework can make the model learn the PET knowledge, whereby the model performs tumor segmentation using the learned PET knowledge without the need for real PET images. This method facilitates a more detailed delineation of the tumor boundaries. In addition to incorporating PET knowledge, our tumor area detection method is also beneficial in enhancing overall performance. Future work will focus on further refining the model and validating its performance on larger and more diverse datasets.

## Data availability statement

Publicly available datasets were analyzed in this study. This data can be found here: NSCLC+Radiomics (NSCLC+Rad, updated 2021/06/01) https://www.cancerimagingarchive.net/collection/nsclc-radiogenomics/, NSCLC-Radiomics (NSCLC-Rad, updated 2020/10/22) https://www.cancerimagingarchive.net/collection/nsclc-radiomics/, NSCLC-Radiomics-Interobserver1 (NSCLCRad-Int, updated 2020/08/13), https://www.cancerimagingarchive.net/collection/nsclc-radiomics-interobserver1/, and Medical Segmentation Decathlon Task06 (MSD Task06) http://medicaldecathlon.com/dataaws/.

## Ethics statement

Ethical approval was not required for the study involving humans in accordance with the local legislation and institutional requirements. Written informed consent to participate in this study was not required from the participants or the participants' legal guardians/next of kin in accordance with the national legislation and the institutional requirements.

## Author contributions

LZ: Funding acquisition, Conceptualization, Formal analysis, Investigation, Methodology, Project administration, Software, Supervision, Validation, Writing – original draft, Writing – review & editing. CW: Data curation, Formal analysis, Validation, Visualization, Writing – original draft, Writing – review & editing. YC: Formal analysis, Methodology, Validation, Writing – review & editing. ZZ: Conceptualization, Resources, Supervision, Validation, Writing – review & editing.
